# RESTAURA project: Using public participation GIS to explore technological risk, landscape, and health perceptions in Camp de Tarragona

**DOI:** 10.1016/j.mex.2026.103904

**Published:** 2026-04-10

**Authors:** Mahdi Gheitasi, Yolanda Pérez Albert, David Serrano Giné

**Affiliations:** a GRATET Research Group, Department of Geography, Universitat Rovira i Virgili, 43480 Vila-Seca, Spain; b Department of Geography, Universidad Nacional de Educación a Distancia, 08016 Barcelona, Spain

**Keywords:** PPGIS, Public perception, Spatial survey

## Abstract

The RESTAURA project proposes an integrated public participation GIS (PPGIS) methodology to investigate the relationships among technological risk perceptions, landscape perception, and self-reported mental health in petrochemical clusters in Camp de Tarragona. The method combines spatial and non-spatial data through PPGIS platfrom to capture both the geographic distribution of perceived risks and the subjective experiences of affected communities. The methodology follows a four-phase deductive mixed-methods design to allow integration of participatory inputs with spatial analysis to support comparative and transferable insights. The approach emphasizes the role of public participation in identifying perceived risk sources, affected areas, and emotionally valued or stressful landscapes. By linking spatial perceptions of technological risk with landscape evaluations and perceived stress, the method provides a comprehensive framework for examining how industrial environments influence well-being. The methodology is designed to be transferable to other industrial urban contexts and to support planning and policy debates on risk reduction, urban design, and public health.

• Collection of spatially explicit public perceptions of technological risk, landscape values, and health using a online survey.
• A combined spatial and statistical analysis to explore the relationship between perceived risk, landscape features, and mental health outcomes by using ArcGIS Pro 3.3.0, InVEST 3.14.3 and Jamovi 2.6.17.

## Graphical abstract

**Figure:**
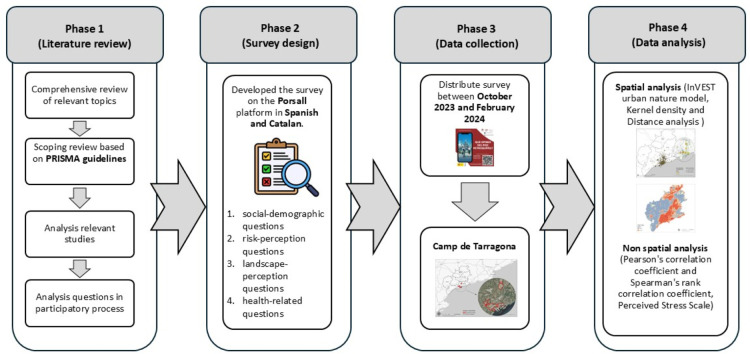

## Specifications table

**Table:** 

**Subject area**	Environmental Science
**More specific subject area**	Geography, Spatial planning
**Name of your method**	PPGIS Survey Workflow for Assessing Technological Risk, Landscape, and Health Perceptions
**Name and reference of original method**	N/A
**Resource availability**	Porsall platform, ArcGIS Pro 3.3.0, InVEST 3.14.3, Microsoft Excel, Jamovi 2.6.17, Adobe Photoshop CC 2020 (v21.1.2.136, Windows), Geospatial and non-spatial datasets (https://hdl.handle.net/20.500.11797/PC4456)

## Background

Industrialization has undergone significant changes and progress since its inception in the 18th century [1]. As the petrochemical industry expands, so do the environmental and human health concerns associated with oil refining and chemical processing [2]. The petrochemical industry and its associated effects, including heavy noise and explosions, have a significant impact on the local population, reinforcing the need for tools capable of capturing how affected communities perceive and spatially locate these risks. Therefore, people who live near technical installations or are exposed to technology-induced hazards are seriously concerned about potential adverse side effects [3,4], and it is necessary to understand their perceptions.

Traditionally, risk perceptions and their impacts on health and the environment are assessed through participatory processes, such as questionnaires and paper-based surveys. However, emerging information technologies and social media have shifted research toward public perceptions of technological risks arising from industrial complexes. The appearance of digital platforms offering new participatory channels allows innovative methodologies such as PPGIS. PPGIS is considered a field within Geographic Information Science (GIS), where citizens can utilize geospatial technologies to generate georeferenced perception data that supports decision-making processes [5].

The motivation for developing and documenting this methodology arises from three research gaps. First, the rapid expansion of the petrochemical and industrial sectors highlights the need for closer examination of their environmental and health impacts. In particular, understanding how risk perception mediates these impacts is essential for informing policy measures aimed at mitigating adverse health outcomes.

Second, the assessment of risk perceptions and impacts was conducted using traditional participatory methods, including interviews and paper surveys. Limited studies have therefore been carried out using emerging technologies for participatory processes, such as PPGIS. As a spatial data collection tool, PPGIS incorporates local knowledge, as well as the perceptions and experiences of residents and stakeholders, which are often lacking in traditional methods. It helps to collect georeferenced information from non-specialist users [6], combine survey data with mapped information, and map and analyze the vulnerability of a wide range of landscape and environmental values [7], as well as values regarding socio-demographic characteristics of the population [6].

Third, understanding the link between the urban environment and health, particularly the benefits of outdoor spaces for mental health, is of growing importance [[7], [8], [9]]. While the positive effects of green spaces and parks have been widely studied, research on the impact of industrial environments on mental health, particularly in relation to stress, remains scarce. In this context, the effect of technological risk on mental health has received little attention. This is why urban planners and architects must consider how different types of built environments, including both natural and industrial, influence stress levels and overall mental well-being. However, to the best of our knowledge, no studies have proposed methodological frameworks capable of analyzing risk perception from a spatial, landscape, and perceived stress perspective.

This method describes how spatial perceptions of technological risks and affected areas can be collected, processed, and analyzed using participatory mapping techniques, and how these perceptions can be linked to perceptions of landscapes and health. By describing the design, implementation, and analysis steps in detail, this methodological paper provides a practical framework that can be adapted for use in other industrial urban environments and applied independently of the original case study.

## Method details

### Case study description

**Fig. 1:**
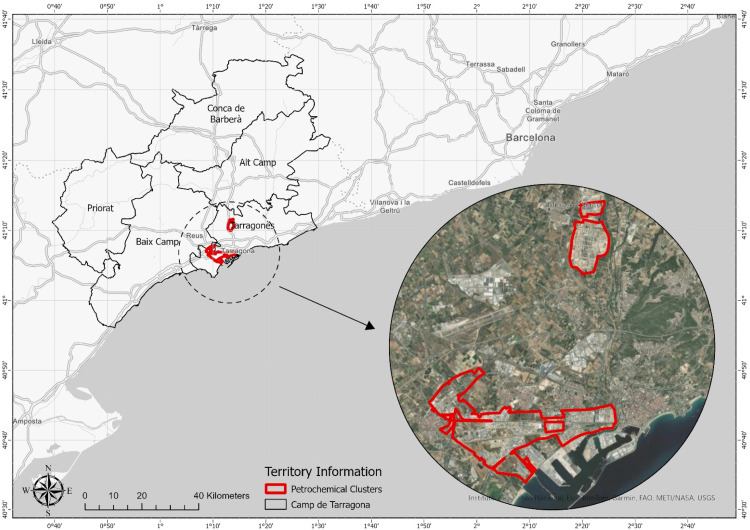
Tarragona petrochemical cluster's location.

source: Earthstar geographics.

The Tarragona petrochemical area is densely populated and is home to important chemical and petrochemical industries. They cover an area of 1500 hectares and contain approximately 30 chemical and petrochemical companies, concentrated in two industrial zones: Polígono Sur (South Industrial Zone) and Pológono Norte (North Industrial Zone), which are approximately 10 km apart from each other [10] (Fig. 1). Polígono Sur (built in 1965–1970), the site nearest the centre of Tarragona, is located near the sea, covers 720 hectares divided between the municipalities of Tarragona, Vila-Seca, and Reus, and the land is owned by the port authority of Tarragona [10]. In addition to the above-mentioned sectors, the area also contains the loading and unloading terminals used by the chemical companies in the port of Tarragona, from which a series of pipelines (crosstabs) transport raw materials and products needed and produced by the companies, and which provide a link between the two industrial sites [10].

In the meantime, Polígono Norte (built in 1971–1980) covers 770 hectares, contains a large oil refinery, and is located in the municipalities of La Pobla de Mafumet, Morell, Perafort, and Constantí, along the main highway N-240.

Approximately 20 million tons of products, mostly oil derivatives, are processed each year, including fuel, asphalt, glue, household gas, lubricants, and textile fibres [12]. According to 2005 data, chemical companies create approximately 30,000 jobs, of which around 6000 are provided directly by the companies themselves, and the rest are provided indirectly or through related activities [11].

### Methodological phases

This method combines qualitative and quantitative elements to provide a comprehensive overview of the research challenge as it unfolds. The qualitative component informed the formulation of the survey questions, ensuring that respondents' perceptions and experiences were accurately captured. The quantitative component made it easier to measure and analyze the responses, as well as compare findings across groups and contexts. The deductive approach forms the basis of research design, as existing theories and concepts guide the operationalization of questions and the testing of hypotheses through the analysis of a case study. This approach ensures that the methodology aligns with the research objectives, encourages the collection of both descriptive and analytical data, and allows for the integration of various forms of evidence to reinforce the overall conclusions.

This method is structured into four main phases, each designed to provide a systematic and consistent approach to understanding perceptual landscapes, technological risks, and mental health.

The method begins (Phase one) with a scoping review, conducted in accordance with the PRISMA-SCR protocol, to identify common approaches and survey questions used in participatory processes for assessing technological risk perception.

The next phase (Phase two) focuses on PPGIS survey questions and the development and implementation of the PPGIS platform, including the adaptation of the interface to support multilingual surveys (Spanish and Catalan) and the possibility of mapping with both single and multiple options. This is followed by the distribution of the questionnaire (phase three), which is carried out through social media, e-mail, and QR-code posters to maximize participation across the case study.

Finally, the data analysis techniques (phase four) combine spatial analysis in ArcGIS Pro 3.3.0 and InVEST 3.14.3 with non-spatial statistical analysis in Microsoft Excel and Jamovi 2.6.17, integrating geographic patterns with socio-demographic, landscape, and health-related insights.

ArcGIS Pro 3.3.0 is a specific minor version release of ArcGIS Pro, a full-featured professional desktop GIS application developed by ESRI. The InVEST model is an open-source software suite for modelling ecosystem services (ESs), provided by Natural Capital Projects and Stanford University [12]. Jamovi 2.6.17 offers basic functions, including data entry and manipulation, rule-based data filtering, variable transformation, and variable calculation. Jamovi 2.6.17 can perform many types of single-variable and multi-variable analyses. These include descriptive statistics, *t*-tests, ANOVA, ANCOVA, MANCOVA, linear regression, exploratory and confirmatory factor analysis, and nonparametric tests [13].

#### Phase one^1^

A scoping review protocol was developed based on the PRISMA-SCR.^2^ It was chosen because scoping reviews focus on topics where different study designs may be used, which requires a comprehensive systematic review of the existing literature. Peer-reviewed papers that investigated the linkages between the public participatory process and perceptions of petrochemical risk, published from January 2000 to December 2022, were included for review. For a paper to be included in the review, it had to meet the following criteria:

• It should apply a public participatory process (e.g., PPGIS, Participatory mapping, questionnaire).
• It should focus on the perception of technological risk.
• It should focus on the petrochemical industry, or other synonymous.
• It should be written in the English language.

Papers meeting the inclusion criteria were identified in 3 steps. Firstly, 1) a literature search was conducted on the Web of Science (WOS) and SCOPUS using search terms synthesized in search sentences (Supplementary materials). The four key terms were public participation, geographical information system, risk perception, and petrochemical; a set of synonyms was chosen for each term (Supplementary materials). Afterwards, 2) a prospective search was made in Google Scholar using a sentence with selected keywords used in the papers' abstracts (perception of risk, technological site, public participation, GIS). Finally, 3) the inclusion/exclusion criteria were applied.

**Fig. 2:**
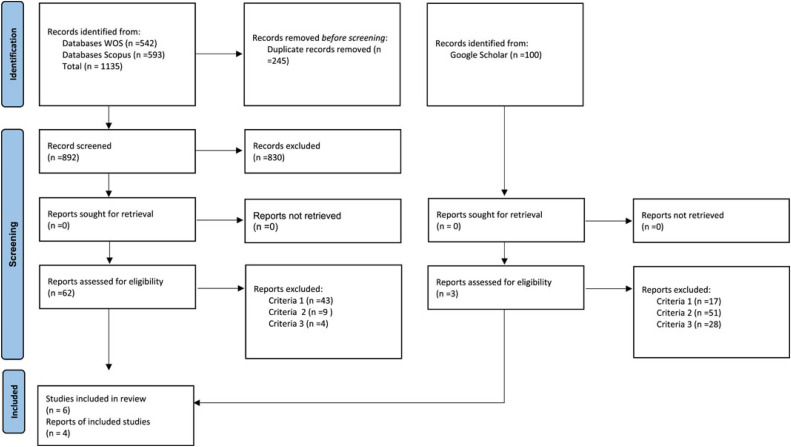
PRISMA 2020 flow diagram for new systematic reviews, which included searches of databases, registers, and other resources.

These searches were done in three databases. Initially, 1135 articles were identified on WOS and SCOPUS; these were exported into reference management software, Zotero, and duplicates were removed, resulting in 892 unique articles. After an abstract screening, 62 articles were identified. A full-text screening identified six articles that met the inclusion criteria. Second, in addition to using the WOS and SCOPUS databases, Google Scholar was employed as an external database to enhance the inclusion of relevant scholarly articles. Given the numerous Google Scholar results (over 100,000 entries, including grey literature), only the first 100 articles from each search were screened, according to a study by Haddaway et al. (2015) [14]. Six papers fulfilled all established inclusion criteria. Additionally, a review of the Google Scholar database identified 100 papers, of which four were selected (Fig. 2).

#### Phase two

Implementing the proposed architecture for PPGIS requires an interface that allows people to interact with and enter data into the platform. Therefore, the survey was created in eight pages, written in Spanish and Catalan, to encourage participation. It was designed to be conducted online using the Porsall platform (https://porsall.com/), facilitating efficient and organized data collection.

**Fig. 3:**
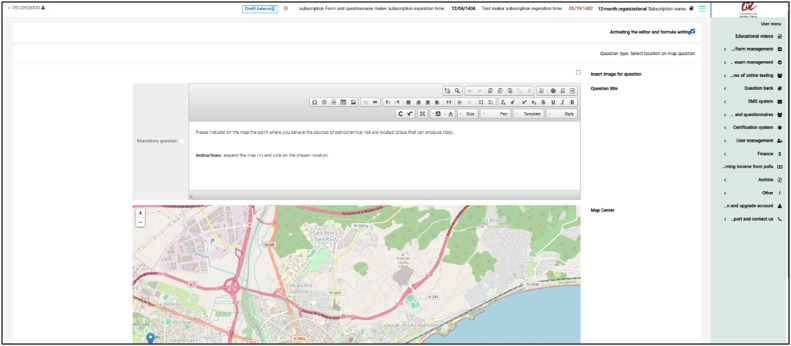
Adapted platform menu.

The platform has been tailored to align with the research's aims and objectives. In the original version, participants could choose only one point per question on a map. To meet the needs of research, the platform has been adapted (modifying the platform's standard code) to allow multiple-choice mapping questions, allowing participants to express their views more clearly (Fig. 3). Additionally, logos and information from the university and project have been integrated into the platform to enhance participants' trust. The first development and implementation of PPGIS occurred in May 2023, with the Farsi-language version of the survey questions serving as the initial data collection and validation for the Tehran Oil Refinery case study. The second development and implementation phase for the Camp de Tarragona case study took place between October 2023 and February 2024, covering both the Spanish and Catalan versions.

**Fig. 4:**
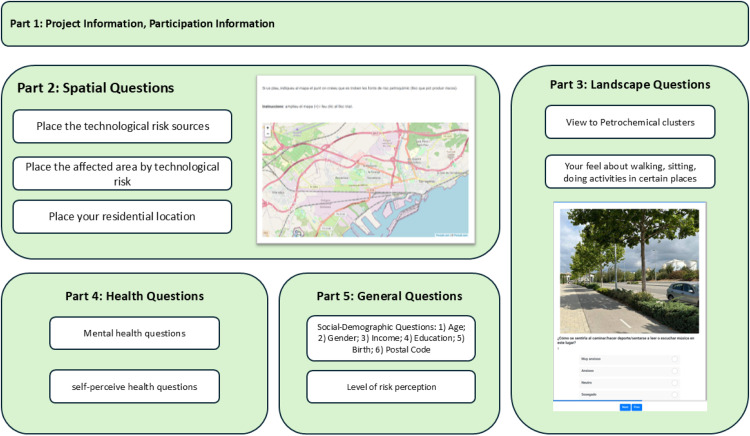
Overview of the questions list in the PPGIS questionnaire.

Survey questions have been developed based on the scoping review results and have been analyzed and adjusted to align with the objectives. The PPGIS survey consisted of six social-demographic questions, four risk-perception questions, two landscape-perception questions, and five health-related questions. Fig. 4 shows the structure of each section and the questions (full description of the questions is available in the Supplementary materials).

The first part of the survey covers the project, participant details, and ethical aspects. The second part consists of questions regarding the location of the risk sources, places affected by risk sources, and the participants' residential locations. In this section, participants were asked to select potential locations by marking points (a maximum of 10 points per question).

In the third section of the survey, participants were asked to express their perceptions of landscape values using both original and edited photographs. This section is based on a previous work conducted by Svobodova et al. (2014) [15], in which the authors selected pictures based on the visibility of the oil refinery and people's awareness. To capture perceptions, participants assessed the photographs using a set of semantic adjective pairs (Table 2). These pairs were drawn from a comprehensive list of adjectives known to influence landscape perception, with three groups selected as the most relevant to the project's objectives. The selected adjectives have been arranged in pairs of adjectives with opposite meanings, in line with the approach of Perovic and Folic (2012) [16].

The photographs were taken from three places in Camp de Tarragona. The photographs were digitally manipulated in two ways: (1) to reduce the visual presence of the refinery and industrial infrastructures and (2) to introduce other negative industrial features, thereby reinforcing the perceived impact of refineries and industrial areas. Based on these photographs, participants were then asked to reflect on how they would feel if they were to engage in daily activities, such as sports, reading, or walking, in such a landscape. In addition to these perceptual assessments, participants answered questions about their experience of seeing the petrochemical or oil refinery from their home or workplace. The photographs were manipulated using Adobe Photoshop CC 2020 (v21.1.2.136, Windows). Initially, the manipulations involved adding elements such as flower boxes and benches. However, after discussions within the research group, it was decided only to add green elements. This approach aimed to reduce the visual impact of negative elements in the landscape without altering the viewer's perception by making the scene appear different (Table 1).

**Table 1:** Approved and disapproved manipulated photographs of the selected landscapes.

Original photograph	Not approved manipulation	Approved manipulation

**Table 2:** Adjective description, source: [17].

Adjectives	Description	Adjectives	Description
Anxious	Feeling or showing worry, nervousness, or unease about something with an uncertain outcome.	Serene	Peaceful and calm; worried by nothing.
Restless	Unwilling or unable to stay still or to be quiet and calm, because you are worried or bored.	Tranquil	Calm and peaceful and without noise, violence, and worry.
Tense	Nervous and worried and unable to relax.	Calm	Peaceful, quiet, and without worry.

In the fourth section, the participants were first asked about their overall health in the last year. For the overall health question, participants were asked to rate their health on a Likert scale (1 to 5), ranging from very bad to very good. Secondly, participants were asked questions about their mental health, specifically exploring how they perceived stress arising from the presence of oil refineries and petrochemical clusters. Therefore, participants were asked how frequently they experienced certain feelings or thoughts; each question was treated individually. The questions included:

1. How often have you felt that you were unable to control the important things in your life?
2. How often have you felt confident about your ability to handle your personal problems?
3. How often have you felt that things were going your way?
4. How often have you felt difficulties piling up so high that you could not overcome them?

For the mental health questions, participants indicated the frequency of specific experiences by selecting one of the following options: never, almost never, occasionally, often, or very often. These options were designed based on the Perceived Stress Scale (PSS) provided by Cohen et al. (1983) [18]. In this method, the short version of the scale, known as the PSS-4 [19], was used.

Finally, section five of the survey focused on socio-demographic questions. This section included the participants' age, educational level, income, and other fundamental characteristics.

#### Phase three

**Fig. 5:**
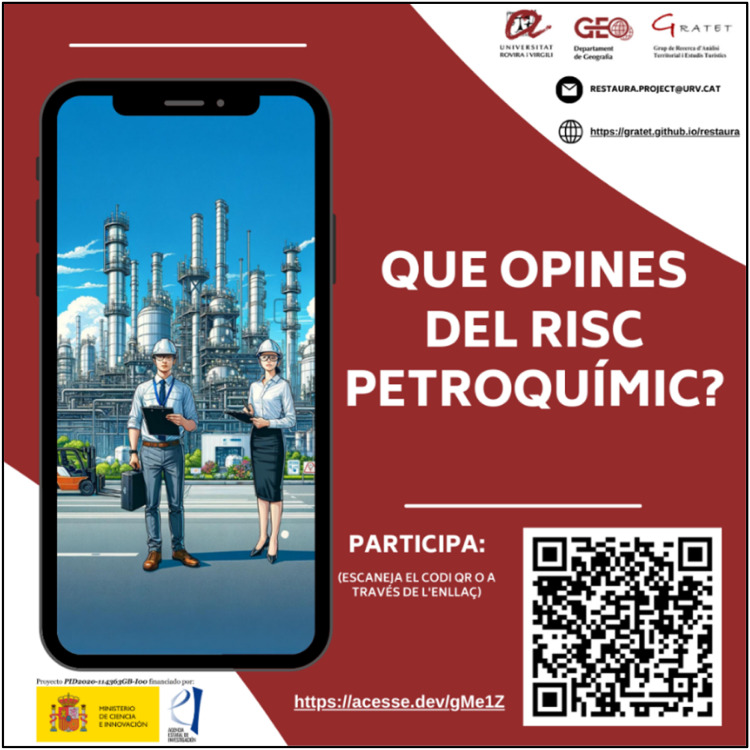
The survey poster in Camp de Tarragona.

In addition to disseminating the survey via social media channels such as Telegram and WhatsApp, e-mails were sent to the university community and neighborhood associations, inviting them to participate. A survey poster with a QR code was created for distribution to the public to promote the survey further (Fig. 5). The survey was also shared on LinkedIn as a post in English, Spanish, and Catalan on the author's profile to generate more reactions. In addition, to ensure a balanced distribution of responses across different areas, phone calls were made to neighborhood associations in those areas with fewer responses, requesting that they send the survey to their members.

#### Phase four

In this phase, three types of analyses were conducted. First, spatial data analysis was performed using the PPGIS data. This included kernel density analysis of participants' locations and levels of risk, analysis of selected points from the map-based survey questions, as well as distance analysis between green spaces and the locations of technological risk sources identified by participants. Second, the InVEST analysis was carried out to identify spatial patterns related to urban nature access. Third, non-spatial analysis of the PPGIS data was conducted, including correlation and regression analyses between socio-demographic variables, levels of risk, and selected spatial data. The details of each part are explained below.

### General spatial data analysis

Before analyzing the spatial data obtained from the PPGIS survey, all responses located outside the case study boundaries were cleaned to ensure accuracy. This involved correcting or discarding inaccurate spatial records and reorganizing the dataset for analysis. In particular, responses from outside the Camp de Tarragona have been excluded; the Clip tool in ArcGIS Pro 3.3.0 has been used to ensure spatial accuracy.

**Fig. 6:**
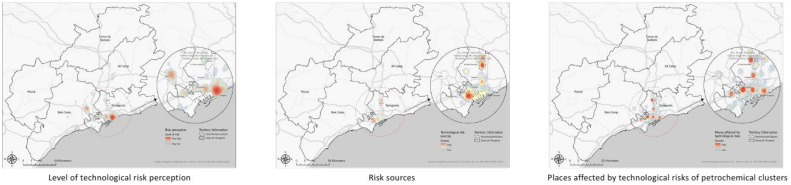
Spatial data analysis maps in Camp de Tarragona.

After cleaning the data, the dataset is ready for spatial analysis. The spatial dataset included the locations of participants' residences (identified separately on the survey map), as well as the locations of risk sources and the areas they considered affected by these risks. Spatial data were associated with additional variables, including socio-demographic characteristics, landscape values, and self-reported health information. To analyze the spatial data, kernel density analysis was performed (Fig. 6). The analysis was conducted in ArcGIS Pro 3.3.0. This approach builds on previous research related to risk perception [[20], [21], [22]].

A distance analysis of green areas in Camp de Tarragona was also conducted using ArcGIS Pro 3.3.0. Distance analysis calculates the distance to the cheapest source. To determine the distance between green areas and the technological risk sources identified by participants, the Near tool in ArcGIS Pro 3.3.0 was used. The Near tool calculates the distance between the input features (points) and the closest feature in another layer (green spaces polygons).

### InVEST model analysis

Additionally, the InVEST model (software InVEST 3.14.3) was utilized to identify spatial patterns related to urban nature access. The model aims to support decision-makers by assessing the trade-offs between alternative management options in the context of ESs and by identifying areas where investments in nature can contribute to human development and the protection of nature. InVEST models combine land use and land cover data with other information, such as soil type and climate, to produce outputs for the biophysical or economic units of the ESs.

The InVEST model calculates the difference between the urban nature provided to a pixel and the population's needs within that pixel. Population need refers to the demand for access to urban nature, especially green spaces. It is assumed that access to urban nature can be linked to a lower level of risk or that risk perception is associated with minimal impact. The InVEST urban nature access model was chosen over other InVEST models because it explicitly calculates access to nature and is therefore suitable for investigating the proposed link between equal access and mitigation of technological risk perception. The INVEST urban nature access model provides a measure of both supply and demand for urban nature and a balance between supply and demand, quantifying the degree to which supply meets demand at individual, municipal and community level [23]. A full description of the model and mathematical expressions can be found on the Natural Capital Project website.^3^

**Fig. 7:**
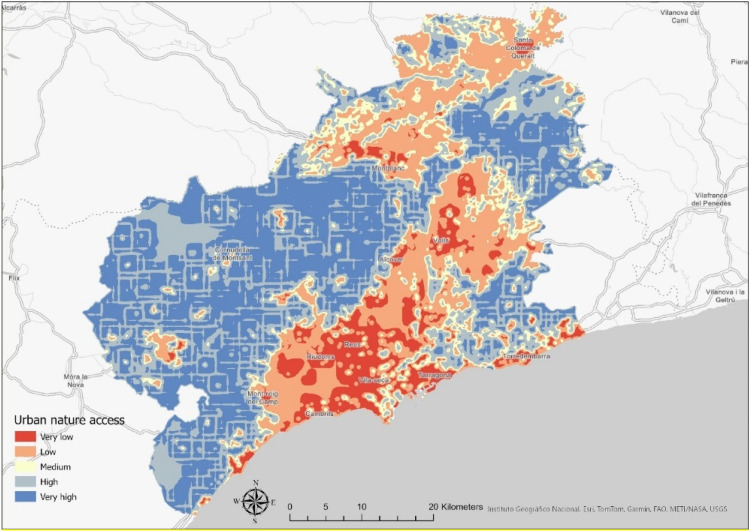
Urban nature access using the InVEST model of the Camp de Tarragona.

Input parameters were utilized in the InVEST model, including a land use and land cover (LULC) raster,^4^ in which each category was assigned a unique integer code corresponding to the LULC attribute table. This table identifies which LULC codes represent urban nature, and a gridded population data representing the population per pixel. Another input parameter is the minimum amount of urban nature required, which is equal to 9 m² per capita, as stated in WHO (2010)[24]. The model's output (continuous raster data) was reclassified into five quantile-based categories (very low, low, medium, high, and very high) to transform numerical values into meaningful, actionable qualitative classes (Fig. 7).

After creating the Urban Nature Access Model, the raster file was imported into ArcGIS Pro 3.3.0, and urban nature data (continuous raster data) was extracted using the Extract Multi-Value to Points tool in ArcGIS Pro 3.3.0 to define an access value for each point location influenced by technological risk. This process resulted in an attribute table where each point possessed a corresponding urban nature access value. This spatial data was joined with survey data (level of risk, age, level of income, and education) to facilitate correlation between them. Consequently, each point in the final dataset contained specific attributes, including urban nature access value and information of the participant who selected the point (such as age, education level, and risk level).

### Non-spatial data analysis

Similarly, for spatial data, all non-spatial data collected in the PPGIS survey were first reviewed, and all responses located outside the case study boundaries were cleaned to ensure accuracy. The non-spatial dataset consisted of three sections: socio-demographic information, picture-based questions (landscape), and health-related questions.

For the socio-demographic section, percentages were calculated for each category (e.g., age, education level, income level, gender) to reflect the proportion of total respondents. Additionally, statistical analysis was performed using Excel for descriptive summaries and Jamovi 2.6.17 for further statistical exploration and visualization.

For the picture-based questions, data extracted from the landscape perception section were analyzed to reflect the proportion of total respondents. Each original and manipulated photograph was evaluated for emotional content using three bipolar semantic scales: Anxious/Serene, Restless/Tranquil, and Tense/Calm. Percentages were then calculated to represent the proportion of respondents assigning a specific emotion (e.g., Anxious, Restless, Tense) or its opposite (Serene, Tranquil, Calm) to each picture. This allowed for a direct comparison between the original and manipulated photographs, demonstrating how the manipulation of the photographs affected emotional perceptions. Analysis was performed using Excel for data aggregation and visualization, and was completed with Jamovi 2.6.17 for statistical testing.

For health-related questions (stress questions), total scores of the PSS were calculated according to the scoring instructions provided in the PSS-4 studies [18,25]. The scale includes four questions that ask participants about their feelings and thoughts during the last month. Respondents indicate how often they experienced each feeling by selecting one of the following options: Never, Almost never, Sometimes, Fairly often, or Very often. Questions 1 and 4 are scored as follows: 0 = Never, 1 = Almost never, 2 = Sometimes, 3 = Fairly often, 4 = Very often. Questions 2 and 3 are reverse-coded, scored as 4 = Never, 3 = Almost never, 2 = Sometimes, 1 = Fairly often, 0 = Very often. The total score was calculated by adding the scores of the four items to provide a total measure of perceived stress. For example, if a participant's responses are as follows:

• Question 1 (PSS-1): Almost Never (score of 1)
• Question 2 (PSS-2): Very Often (score of 0)
• Question 3 (PSS-3): Sometimes (score of 2)
• Question 4 (PSS-4): Very Often (score of 4)

The total score is calculated as: Total Score = Score of PSS-1+Score of PSS-2+Score of PSS-3+Score of PSS-4 = 1 + 0 + 2 + 4 = 7. According to the categorization, a total score of 7 falls into the Medium Stress category. After the total scores are calculated, they are broken down into the following categories.

• Low Stress: 0–4
• Medium Stress: 5–8
• High Stress: 9–12
• Very High Stress: 13–16

Furthermore, a correlation matrix was generated to examine the relationship between the different types of risk-related variables (technological risk perception, landscape perception, and health perception). Both Pearson's correlation coefficient and Spearman's rank correlation coefficient are applied. These analyses were performed using Jamovi 2.6.17 statistical software.

## Method validation

The proposed PPGIS methodology was first applied in the Tehran Oil Refinery case study to assess its operational feasibility, data quality, and analytical compatibility. The survey was successfully deployed in Farsi using a multi-page online interface and complemented by in-person data collection to ensure inclusivity. A total of 220 valid responses were collected. Participants identified 741 georeferenced points representing perceived technological risk sources and 800 points representing areas affected by technological risks after data cleaning. The spatial distribution of mapped risk sources showed a clear concentration in the southern part of Tehran, particularly within and around the oil refinery and its associated facilities, demonstrating the method's ability to capture spatially explicit perceptions aligned with known industrial infrastructures.

The dataset allowed the integration of spatial information with socio-demographic and health-related variables. Stress levels, as calculated using the PSS-Studies, were successfully categorized, with the majority of respondents falling within the medium stress range. The collected data were compatible with kernel density analysis and statistical processing, confirming that the method generates structured and analyzable spatial and non-spatial outputs suitable for participatory risk perception studies. The detailed results and data collected in this study are available in the URV repository.^5^

## Limitations

In the data collection for Camp de Tarragona, the study involved 445 participants. The following reasons can explain this low response rate: 1) lack of time and effort to participate, 2) problems with the technical requirements of online surveys, and 3) level of personal interest in the study context [26,27]. The low response rate affected the capacity to perform spatial statistical analyses. Therefore, the suitable solution is to offer participants different modes of participation, such as paper maps, which will help reduce participant bias and increase mapping participation [26]. Moreover, offering various types of incentives, such as small cash rewards, gift vouchers, or access to study results, can further encourage participation and increase the response rate.

Previous studies [28] have shown concerns regarding the validity of PPGIS data, which are also faced in this project. PPGIS surveys have the potential to reach populations that lack access to GIS technology. Groups commonly face these problems due to lower education and income levels [29]. Additionally, the involvement of non-experts who lack adequate knowledge of spatial awareness, geography, and individual characteristics can occasionally introduce issues with spatial precision in PPGIS studies [28]. By addressing these issues, the use of PPGIS in future research can be optimised to the benefit of society and the environment.

The project also used self-reported stress scales, which can introduce subjective bias and affect the validity of the findings. In addition, using a limited set of photographs (three from Camp de Tarragona) and their manipulated versions to represent the landscape around the refinery provided a focused view. However, it could be extended with a larger set of photographs to improve the comprehensiveness of future studies. In addition, the survey primarily focused on visual effects and did not consider other potential stress factors, such as smell or sound, which can also impact the level of stress and environmental perception. Future studies could incorporate a multi-sensory analysis to provide a more comprehensive understanding of the environment's impact on stress.

Although the surveys distributed to participants included open-ended questions to collect qualitative information, the number of responses was minimal (fewer than 10 for Camp de Tarragona). Consequently, these data have been excluded from the analysis. However, the research is still considered a mixed study that integrates both quantitative and spatial approaches.

## Ethics statements

The study was conducted in accordance with the Declaration of Helsinki and approved by the ETHICAL COMMITTEE CONCERNING RESEARCH INTO PEOPLE, SOCIETY AND THE ENVIRONMENT OF THE UNIVERSITAT ROVIRA I VIRGILI (CEIPSA) (CEIPSA-2021-PR-0026, November 10, 2022).

## CRediT author statement

**Mahdi Gheitasi:** Conceptualization, Methodology, Software, Validation, Formal analysis, Investigation, Resources, Data curation, Writing – original draft, Visualization, Funding acquisition. **Yolanda Pérez Albert:** Conceptualization, Methodology, Writing – review & editing, Supervision, Project administration, Funding acquisition. **David Serrano Giné:** Conceptualization, Methodology, Writing – review & editing, Supervision.

## Related research article

N/A.

## Declaration of interests

The authors declare that they have no known competing financial interests or personal relationships that could have appeared to influence the work reported in this paper.

## Data Availability

Data will be made available on request.

## Appendix Supplementary materials

Image, application 1

## Footnotes

1 The results of the first phase were published in https://doi.org/10.3389/fenvs.2023.1218995

## Footnotes

2 https://www.prisma-statement.org/scoping

3 https://naturalcapitalalliance.stanford.edu/invest/urban-nature-access

4 The LULC was downloaded from https://www.icgc.cat/en/Geoinformation-and-Maps/Maps/Land-cover-map-Catalonia

5 https://repositori.urv.cat/estatic/PC0041/en_PC4457.html
