# Supplementary material for: RESTAURA project: Using public participation GIS to explore technological risk, landscape, and health perceptions in Camp de Tarragona
Source: MethodsX. 2026 Apr 10;16:103904. doi: 10.1016/j.mex.2026.103904 (PMC13191105; doi:10.1016/j.mex.2026.103904)
Supplement: Supplementary file 1 [file mmc1.docx]

**Appendix**

Survey in English

This survey is led by the Department of Geography at URV and is part of the RESTAURA research project (PID2020-114363GB-I00, https://gratet.github.io/restaura/). The main objective is to understand your perception of the risks associated with the petrochemical industry.

Your participation is entirely voluntary. The data you provide will be stored and used by the project while respecting your confidentiality.

This survey is intended for adults, so individuals who do not meet this requirement should not participate.

Please review the participant information sheet and the informed consent form before starting. If you need any clarifications, feel free to contact us at the following email address: restaura.project@urv.cat

This study has been approved by the Ethics Committee for Research Involving People, Society, and the Environment at Universitat Rovira i Virgili (CEIPSA-2021-PR-0026).

Thank you very much for your collaboration.

Do you confirm that you have read the participant information sheet and that you wish to voluntarily take part in this study?

Do you authorize the use of the data from this survey under the conditions outlined in the informed consent form?

1. In the past month, how often have you felt unable to control the important things in your life?

| 1. Never |
| --- |
| 1. Almost never |
| 1. Sometimes |
| 1. Often |
| 1. Very often |

1. In the past month, how often have you felt confident in your ability to handle the personal problems in your life?

| 1. Never |
| --- |
| 1. Almost never |
| 1. Sometimes |
| 1. Often |
| 1. Very often |

1. In the past month, how often have you felt that things are going well?

| 1. Never |
| --- |
| 1. Almost never |
| 1. Sometimes |
| 1. Often |
| 1. Very often |

1. In the past month, how often have you felt that the difficulties are piling up so much that you can't overcome them?

| 1. Never |
| --- |
| 1. Almost never |
| 1. Sometimes |
| 1. Often |
| 1. Very often |

1. Please indicate on the map the location where you believe the sources of petrochemical risk are (places that could pose risks).

Instructions: Zoom in on the map (+) and click on the chosen location.


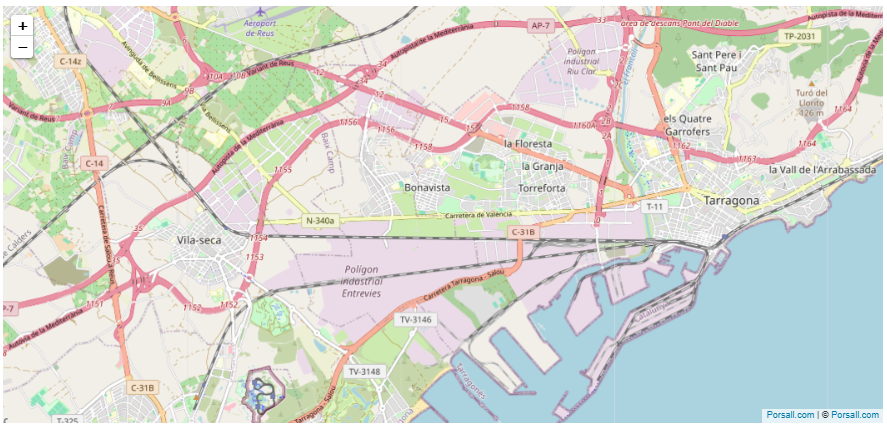


1. Please indicate on the map, if applicable, the locations that you believe could be most affected by a potential petrochemical incident or accident (places affected by the risks).

Instructions: Zoom in on the map (+) and click on the chosen location.


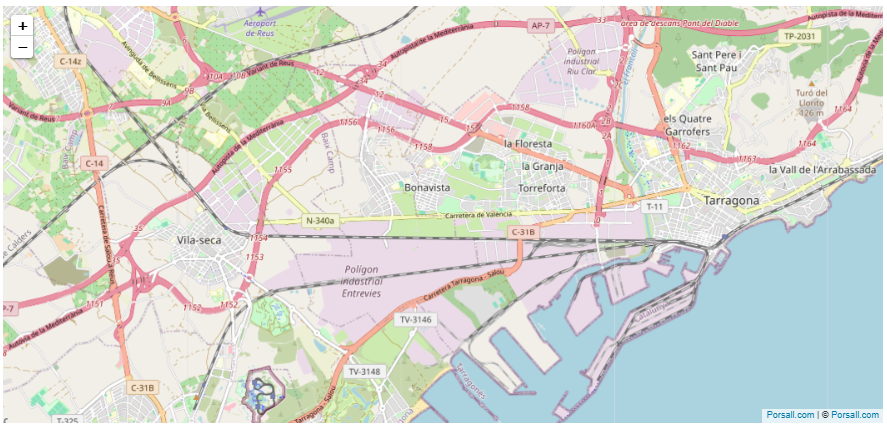


1. ¿What level of risk do you perceive in the petrochemical industry?

- None or very low
- Medium
- High
- Very high

1. Can you see the petrochemical facilities from your home? (from the balcony, terrace, window, etc.)

- Yes
- No
- NS/NC

1. Do you currently work, have you worked, or do you have immediate family members (partner, parent, child, uncle/aunt) working in the petrochemical industry?

- Yes
- No
- NS/NC

Instructions for the landscape questions (photographs): For each photograph, there are three emotions listed, and you need to select the intensity of each emotion (on a five-point scale). You can also add any other emotions the photograph evokes in you by using the last option on the list.

1.
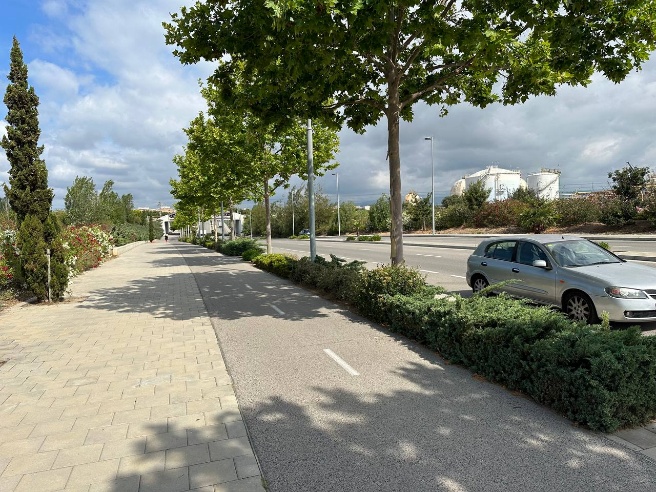
How would you feel walking, exercising, sitting to read, or listening to music in this place?

- Very anxious / Anxious / Neutral / Calm / Very calm
- Very restless / Restless / Neutral / Relaxed / Very relaxed
- Very tense / Tense / Neutral / Calm / Very calm
- If applicable, please indicate other emotions.

1. How would you feel walking, exercising, sitting to read, or listening to music in this place?


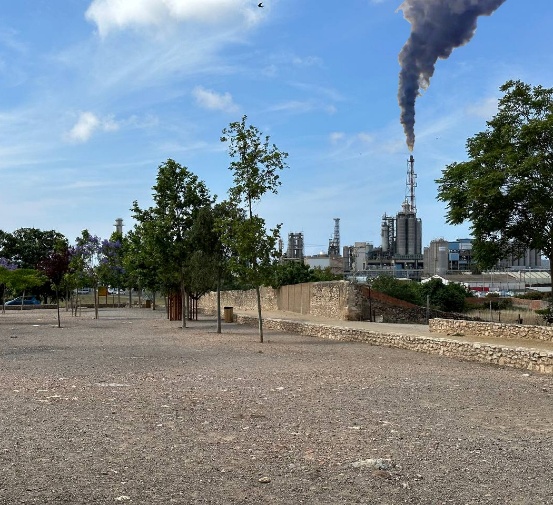


- Very anxious / Anxious / Neutral / Calm / Very calm
- Very restless / Restless / Neutral / Relaxed / Very relaxed
- Very tense / Tense / Neutral / Calm / Very calm
- If applicable, please indicate other emotions.

1. How would you feel walking, exercising, sitting to read, or listening to music in this place?


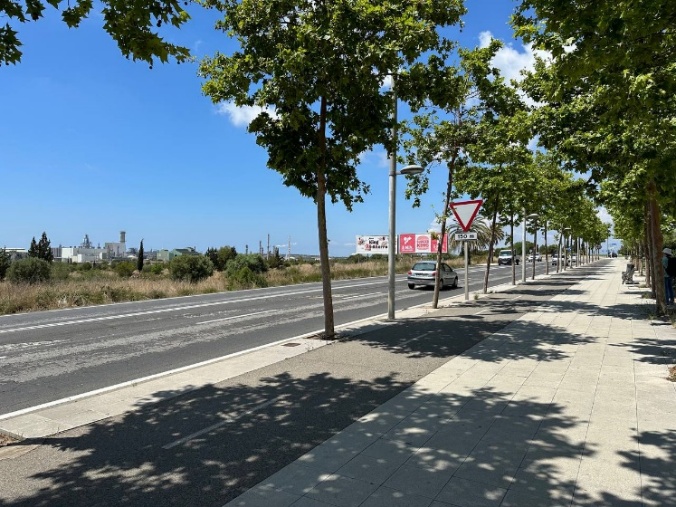


- Very anxious / Anxious / Neutral / Calm / Very calm
- Very restless / Restless / Neutral / Relaxed / Very relaxed
- Very tense / Tense / Neutral / Calm / Very calm
- If applicable, please indicate other emotions.

1. How would you feel walking, exercising, sitting to read, or listening to music in this place?


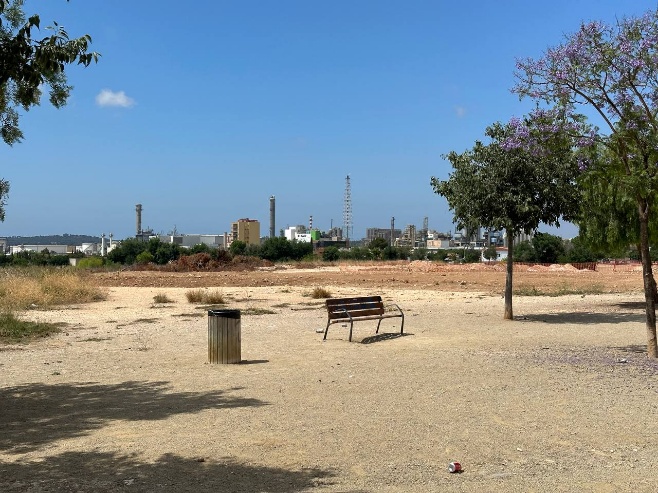


- Very anxious / Anxious / Neutral / Calm / Very calm
- Very restless / Restless / Neutral / Relaxed / Very relaxed
- Very tense / Tense / Neutral / Calm / Very calm
- If applicable, please indicate other emotions.

1. How would you feel walking, exercising, sitting to read, or listening to music in this place?


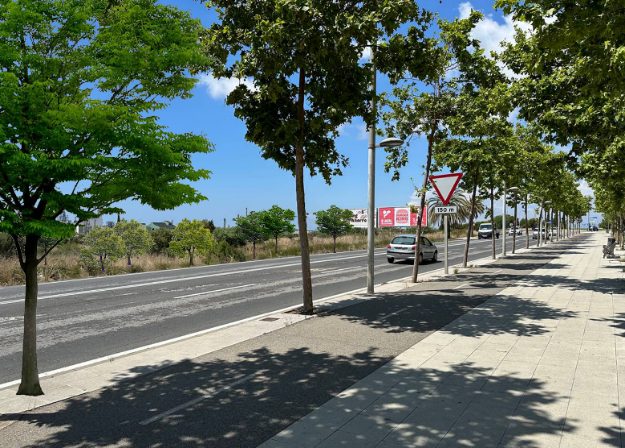


- Very anxious / Anxious / Neutral / Calm / Very calm
- Very restless / Restless / Neutral / Relaxed / Very relaxed
- Very tense / Tense / Neutral / Calm / Very calm
- If applicable, please indicate other emotions.

1. How would you feel walking, exercising, sitting to read, or listening to music in this place?


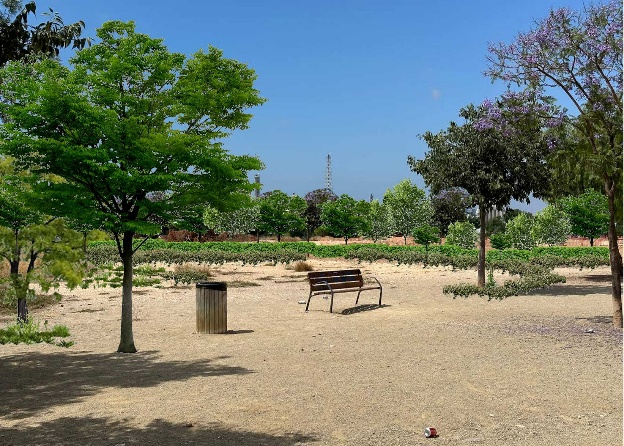


- Very anxious / Anxious / Neutral / Calm / Very calm
- Very restless / Restless / Neutral / Relaxed / Very relaxed
- Very tense / Tense / Neutral / Calm / Very calm
- If applicable, please indicate other emotions.

1. How would you feel walking, exercising, sitting to read, or listening to music in this place?


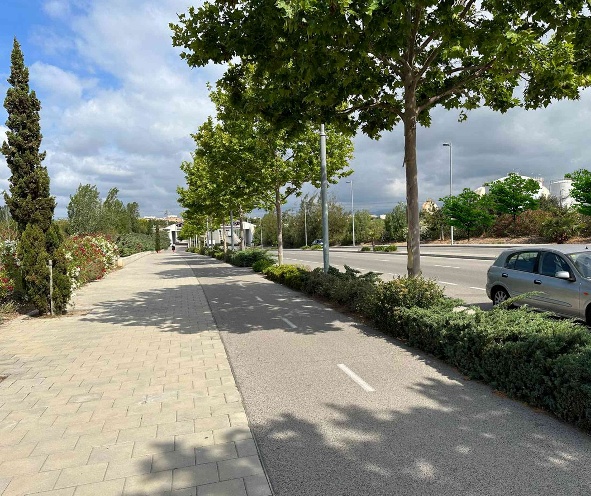


- Very anxious / Anxious / Neutral / Calm / Very calm
- Very restless / Restless / Neutral / Relaxed / Very relaxed
- Very tense / Tense / Neutral / Calm / Very calm
- If applicable, please indicate other emotions.

1. How would you feel walking, exercising, sitting to read, or listening to music in this place?


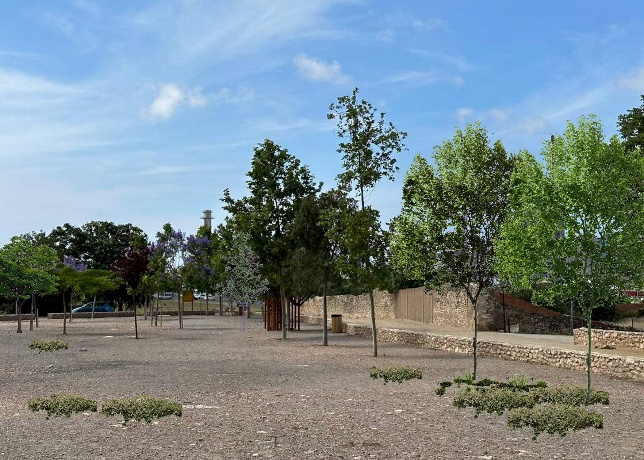


- Very anxious / Anxious / Neutral / Calm / Very calm
- Very restless / Restless / Neutral / Relaxed / Very relaxed
- Very tense / Tense / Neutral / Calm / Very calm
- If applicable, please indicate other emotions.

1. Age:
2. Sex:

- Male
- Female
- Other
- NS/NC

1. Postal code:
2. If you wish, please indicate your approximate place of residence on the map (this information will only be used to calculate the distance to the risk sources):


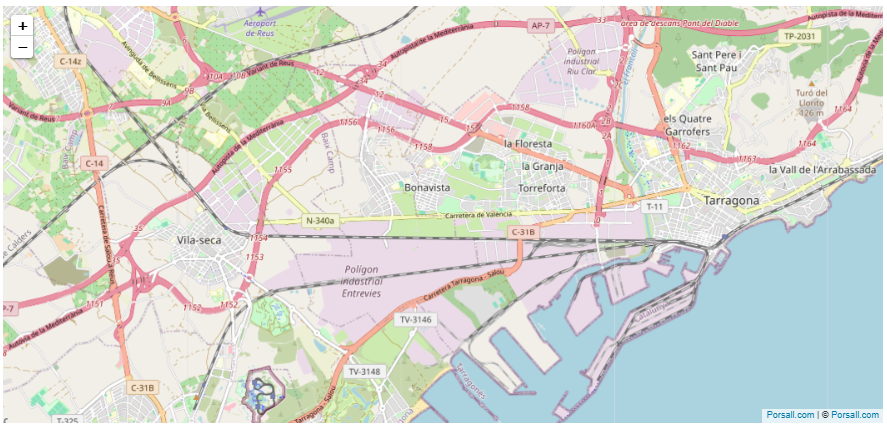


1. ¿What is your average monthly income?

- Without income
- Less than 1.000 €
- Between 1.000 and 1.999 €
- Between 2.000 and 2.999 €
- More than 3.000 €

1. What is your highest level of education completed?

- Without studies
- Primary education
- Secondary education (ESO / High School)
- Vocational training
- University studies
- NS/NC

Search syntax used for literature search.

| **Database** | **Search status** | **syntax** |
| --- | --- | --- |
| **SCOPUS** | Title  Abstract  Keywords | TITLE-ABS-KEY ( “Geographic information system”  OR  “GIS”  OR  “geographic information system for public participation”  OR  “public participation geographic information system”  OR  “PPGIS”  OR  “public participation GIS”  OR  “shared geographic information”  OR  “participatory app”  OR  “participatory GIS”  OR  “PGIS”  OR  “public engagement app”  OR  “public engagement GIS”  OR  “citizen science”  OR  “participatory mapping”  OR  “volunteer geographic information”  OR  “VGI”  OR  “GIS survey”  OR  “geographic information technology”  OR  “GIT” )  AND  TITLE-ABS-KEY ( “technol* “  OR  “manmade “  OR  “man-made “  OR  “anthro* risk” )  AND  TITLE-ABS-KEY ( “risk perception”  OR  “disaster perception”  OR  “catastrophe perception”  OR  “risk assessment”  OR  “disaster assessment”  OR  “catastrophe assessment” ) ) |
| **Web of Science** | Topics | (TS)= ( “Geographic information system” OR “GIS” OR “geographic information system for public participation” OR “public participation geographic information system” OR “PPGIS” OR “public participation GIS” OR “shared geographic information” OR “participatory app” OR “participatory GIS” OR “PGIS” OR “public engagement app” OR “public engagement GIS” OR “citizen science” OR “participatory mapping” OR “volunteer geographic information” OR “VGI” OR “GIS survey” OR “geographic information technology” OR “GIT” ) AND (TS)= ( “technol* “ OR “man-made” OR “man-made “ OR “anthro* risk” ) AND(TS)= ( “risk perception” OR “disaster perception” OR “catastrophe perception” OR “risk assessment” OR “disaster assessment” OR “catastrophe assessment” ) |
| **Google Scholar** | Abstract keywords | “perception of risk” OR “technological site” OR “public participation” OR, “GIS |

Search terminology in WOS, Scopus, and Google Scholar

|  | **PPGIS** | **Petrochemical risk** | **Risk perception** |
| --- | --- | --- | --- |
| **Synonyms 1** | GIS | Man-made risk | Risk perception |
| **Synonyms 2** | Geographic information system for public participation | Anthropic risk | Disaster perception |
| **Synonyms 3** | Public participation geographic information system | Technological risk | Catastrophe perception |
| **Synonyms 4** | PPGIS |  | Risk assessment |
| **Synonyms 5** | Public participation GIS |  | Disaster assessment |
| **Synonyms 6** | Shared geographic information |  | Catastrophe assessment |
| **Synonyms 7** | Participatory app |  |  |
| **Synonyms 8** | Participatory GIS |  |  |
| **Synonyms 9** | PGIS |  |  |
| **Synonyms 10** | Public engagement app |  |  |
| **Synonyms 11** | Public engagement GIS |  |  |
| **Synonyms 12** | Citizen science |  |  |
| **Synonyms 13** | Participatory mapping |  |  |
| **Synonyms 14** | Volunteer geographic information |  |  |
| **Synonyms 15** | VGI |  |  |
| **Synonyms 16** | GIS survey |  |  |
| **Synonyms 17** | Geographic information technology |  |  |
| **Synonyms 18** | GIT |  |  |
